# Effect of Dynamically Arrested Domains on the Phase Behavior, Linear Viscoelasticity and Microstructure of Hyaluronic Acid – Chitosan Complex Coacervates

**DOI:** 10.1021/acs.macromol.3c00269

**Published:** 2023-07-18

**Authors:** Julien Es Sayed, Clément Caïto, Abinaya Arunachalam, Armin Amirsadeghi, Larissa van Westerveld, Denise Maret, Roshan Akdar Mohamed Yunus, Eleonora Calicchia, Olivia Dittberner, Giuseppe Portale, Daniele Parisi, Marleen Kamperman

**Affiliations:** † Zernike Institute for Advanced Materials (ZIAM), University of Groningen, Nijenborgh 4, 9747 AG Groningen, The Netherlands; ‡ Engineering and Technology Institute Groningen (ENTEG), University of Groningen, Nijenborgh 4, 9747 AG Groningen, The Netherlands; § Department of Nanomedicine & Drug Targeting, Groningen Research Institute of Pharmacy, University of Groningen, A. Deusinglaan 1, 9713 AV Groningen, The Netherlands

## Abstract

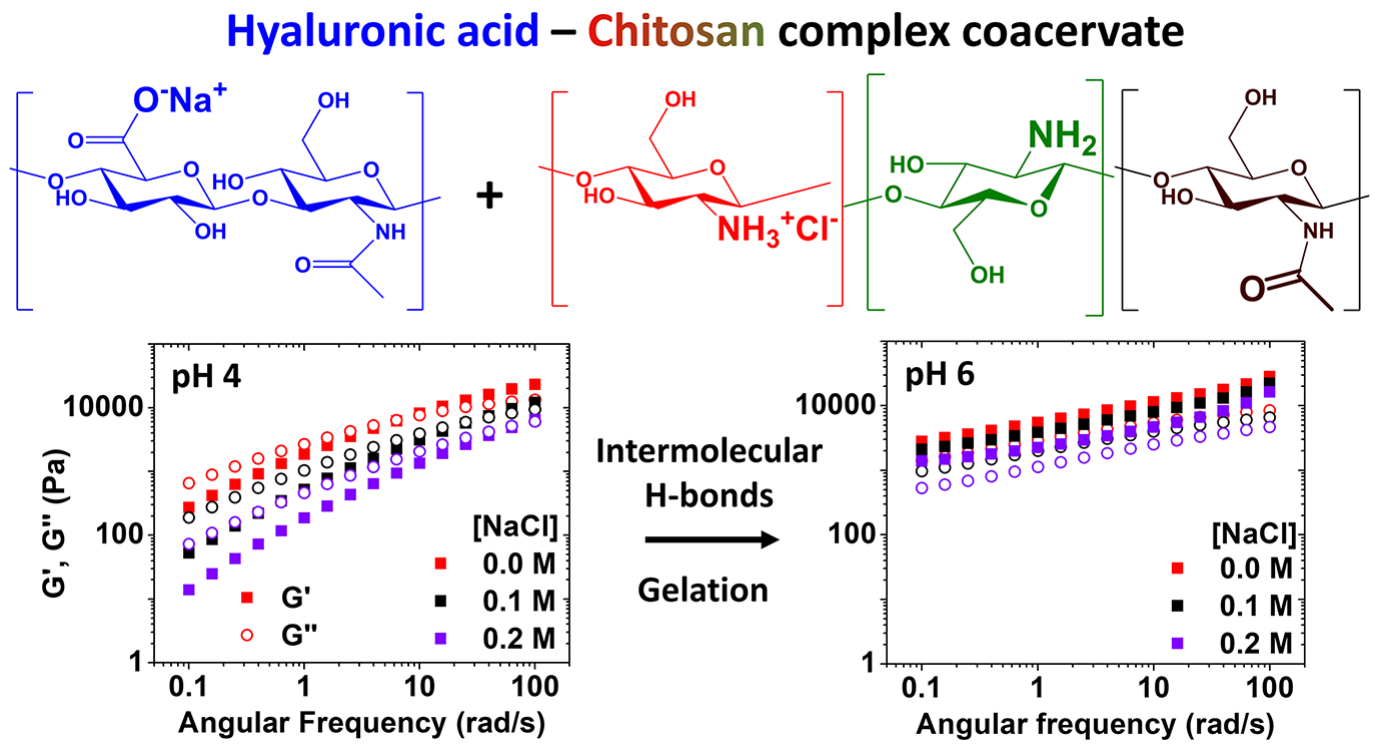

Complex coacervates make up a class of versatile materials formed as a result of the electrostatic associations between oppositely charged polyelectrolytes. It is well-known that the viscoelastic properties of these materials can be easily altered with the ionic strength of the medium, resulting in a range of materials from free-flowing liquids to gel-like solids. However, in addition to electrostatics, several other noncovalent interactions could influence the formation of the coacervate phase depending on the chemical nature of the polymers involved. Here, the importance of intermolecular hydrogen bonds on the phase behavior, microstructure, and viscoelasticity of hyaluronic acid (HA)–chitosan (CHI) complex coacervates is revealed. The density of intermolecular hydrogen bonds between CHI units increases with increasing pH of coacervation, which results in dynamically arrested regions within the complex coacervate, leading to elastic gel-like behavior. This pH-dependent behavior may be very relevant for the controlled solidification of complex coacervates and thus for polyelectrolyte material design.

## 1 Introduction

Complex coacervation is a liquid–liquid phase separation formed through electrostatic interactions between oppositely charged polyelectrolytes and the subsequent release of the originally bound counterions and water molecules.^1,2^ The resulting system consists of a polymer-rich viscoelastic phase, the complex coacervate, in thermodynamic equilibrium with a polymer-poor liquid phase. Complex coacervate materials have found use in a growing number of applications ranging from underwater glues, filtration membranes, fibers, structural materials, 3D printable inks, drug carriers, particulate emulsifiers, and many more.^3−15^

For most of these applications, the fine control over the viscoelastic properties and the composition of the material is necessary. The presence of salt in the medium has been commonly reported (experimentally and theoretically) to alter the viscoelastic behavior of the material for a broad range of polyelectrolyte pairs.^16−23^ For low salt concentrations, the resulting coacervate generally behaves as a viscoelastic solid with slow dynamics. On the other hand, increasing the salt concentration of the medium decreases the driving force for coacervation, rendering the formulated material softer and with fast dynamics (shorter characteristic relaxation times). Spruijt et al. showed that for such systems that are controlled by electrostatic interactions, there is a direct equivalence between time and salt concentration similar to the equivalence of time and temperature in polymer melts.^16,17^ However, even if electrostatic interactions and counterion release are generally considered to be the main driving interactions for coacervation, the final material composition and properties often depend on additional noncovalent interactions.^24^ For natural polymers such as polysaccharides, which contain a variety of chemical moieties as part of the repetitive units, non-Coulomb interactions become critical parameters that cannot be overlooked. In addition to this, solvent–polymer affinity is also a key parameter of the behavior of complex coacervates at a given pH, salt, and temperature.^25^ Recently, Sun et al. investigated the viscoelastic behavior of hyaluronic acid–chitosan complex coacervates in mixtures of water with a cosolvent, namely ethanol and methanol.^26^ The authors showed that modulation of the average dielectric constant of the mixture could be used to control the strength of the electrostatic interactions and consequently the dynamics of the resulting complex coacervate. Next to effects due to changes in the average dielectric constant, the authors also found a possible additional effect of the solvent–polymer affinity on the final dynamics of the material.

The effect of additional intermolecular interactions on the phase behavior of complex coacervates made with pH-sensitive polyelectrolytes was investigated by Li et al.^27^ In this work, poly(acrylic acid) (pAA) was combined with poly(allylamine hydrochloride) (pAH) at three different pH values: 3, 6.5, and 9. The authors observed a classical evolution of the phase-separated mixture at pH 6.5 and 9, with a transition from flaky to gel-like to liquid-like spherical drops when increasing the salt concentration of the medium. In contrast, the complexes retained a flaky precipitate morphology at pH 3 irrespective of the salt concentration. Building on these observations, the authors pointed to the importance of the relative water-insolubility of the pAA chains at acidic pH for which most of the acrylic acid repetitive units are protonated. They proposed the hypothesis of pAA chains serving as hydrogen bond donors and/or acceptors. Altogether, the results reported in this paper emphasized the strong influence of intermolecular chain associations under bad solvent conditions on the final morphology and composition of the resulting complex coacervate material.

Similar to pAA, chitosan (CHI) exhibits a strong water solubility dependence with pH until becoming fully insoluble at pH values above its p*K*a (around 6.5).^28^ Hyaluronic acid (HA)–CHI complexes have been reported to have diverse applications in the biomedical field, including fibers, scaffolds and highly stretchable hydrogels.^9,29−33^ To rationalize the design of these materials, in a recent study, Kayitmazer et al. systematically investigated the effect of molecular weight and deacetylation degree of the CHI on the viscoelastic behavior of the resulting HA–CHI complex coacervates at pH and salt concentrations close to physiological conditions.^34^ However, the fundamentals related to the effect of CHI solubility as a function of the pH on the viscoelastic properties of the coacervate material still remain barely explored. Understanding and controlling the interactions that play a role in the solubility of complex coacervates are of key importance for their processing and thus for the design of complex coacervate-based materials. More specifically, being able to control the liquid-to-solid transition of complex coacervates by (gradual) changes in pH, salt, or other parameters will enable the formation of novel polyelectrolyte materials. For example, recently, we showed that the 3D printability, and more precisely the shape retention ability, of HA–CHI complex coacervates can be controlled by changing the pH during processing.^10^ We observed a higher extent of shape retention as well as slower dynamics at pH values closer to the p*K*a of the CHI chains and assigned this to the relative insolubility of the chains.

Herein, we systematically investigate the influence of pH and salt concentration on the phase behavior, composition, and viscoelastic behavior of the resulting HA–CHI complex coacervates. The viscoelastic behavior was then related to the microstructure of the coacervates by using rheology and X-ray scattering experiments. We particularly focus on the influence of the water solubility of CHI, or its ability to form intermolecular hydrogen bonds (H-bonds) at pH close to its p*K*a. To better understand the role of these H-bonds, we also investigated hyaluronic acid–quaternized chitosan (HA–qCHI) complex coacervates and HA–CHI systems with added urea, a strong hydrogen bond competitor.

## 2 Experimental Section

### 2.1 Materials

Chitosan (CHI) with a degree of deacetylation of 89% (confirmed by ^1^H NMR) and an average molecular weight of 30 kg/mol, and sodium hyaluronate, referred to as hyaluronic acid (HA), with an average molecular weight of 30–50 kg/mol were both purchased from Glentham Life Sciences Co. (Corsham, United Kingdom) and used as received. Sodium chloride (NaCl), hydrochloric acid (HCl), sodium hydroxide (NaOH), glycidyltrimethylammonium chloride (GTMAC) >90% and silver nitrate (AgNO_3_) were purchased from Sigma-Aldrich (Darmstadt, Germany) and used without any further purification. Acetone >99.5% and methanol, anhydrous, were purchased from Macron Fine Chemicals (Avantor Inc., Radnor, PA, USA). Deionized (DI) water was produced by reverse osmosis (conductivity <10 μS/cm).

### 2.2 Titration of HA and CHI

To determine the degree of ionization of HA and CHI as a function of pH, titrations were performed using 5 mg/mL polymer solutions at 0.1 M NaCl starting from a fully protonated form for both CHI and HA. Aliquots of 0.1 M NaOH solution were added to adjust the pH. The effective apparent p*K*a_HA_ and p*K*a_CHI_ were taken as the pH halfway of the equivalence point and were used to calculate the degree of ionization as a function of pH (Figure S2) according to the following equations:
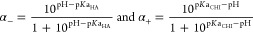
The apparent p*K*a of HA and CHI were then determined to be p*K*a_HA_ = 2.15 and p*K*a_CHI_ = 6.55.

From this, the following degrees of ionization at pH 4 and 6 for both polymers were calculated: α_–,pH4_ = 0.98; α_–,pH6_ = 1; α_+,pH4_ = 1; and α_+,pH6_ = 0.78.

### 2.3 qCHI Synthesis

CHI (4.50 g, 27.1 mmol) was dispersed in 80.0 mL of DI water under stirring at 85 °C for 10 min. GTMAC was added in three aliquots of 3.5 mL (10.5 mL, 78.3 mmol) each after a 90 min interval. The mixture was left stirring for 12 h. The final solution was precipitated twice in 800 mL of a cold 1:6 methanol/acetone mixture. The final powder was oven-dried at 50 °C for 5 h. Finally, the product was solubilized in 100 mL of DI water and further freeze-dried for one night to obtain a white fluffy powder of qCHI (5.12 g).

### 2.4 Measurement of the Degree of Quaternization (DQ)

The degree of quaternization of qCHI was determined via conductometric titration using a 0.017 M AgNO_3_ solution. The evolution of the conductivity of a 1 mg/mL solution of qCHI was measured upon the addition of 0.1 mL aliquots of titrant. As AgNO_3_ was added, the conductivity of the solution decreased due to the precipitation of AgCl (s). Titration was considered complete when all Cl^–^ anions were complexed to Ag^+^ cations, which resulted in an increase in the conductivity of the solution with further addition of titrant.

### 2.5 Calculation of the Charge Ratio for Complex Coacervation

The (−):(+) charge ratio was determined as the ratio  with *n*_–_ and *n*_+_ being, respectively, the total amount of negative and positive repetitive units in the system.

For the HA–CHI complex coacervates, it was calculated as follows:
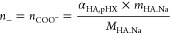

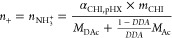
where α_CHI,pHX_ and α_HA,pHX_ are respectively the ionization degree of CHI and HA at the pH X (being 4 or 6), *m*_CHI_ and *m*_HA.Na_ are respectively the total mass of dry CHI and HA.Na in the medium, *M*_DAc_ and *M*_Ac_ are respectively the molecular weight of the CHI deacetylated repetitive units (*M*_DAc_ = 161 g/mol) and acetylated repetitive units (*M*_Ac_= 203 g/mol), *M*_HA.Na_ the molecular weight of the HA.Na repetitive units (*M*_HA.Na_= 401 g/mol), and *DDA* the deacetylation degree of the CHI (*DDA* = 0.89).

For the HA–qCHI complex coacervates, *n*_+_ was calculated as follows:
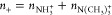

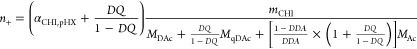
where *M*_qDAc_ is the molecular weight of the CHI quaternized deacetylated repetitive units (*M*_qDAc_= 312 g/mol) and *DQ* the quaternization degree of the CHI (*DQ* = 0.72).

### 2.6 Optimum Complex Coacervation Charge Ratio

The optimum charge ratio for coacervation of the four investigated systems, namely HA-CHI/pH4, HA-CHI/pH6, HA-qCHI/pH4, and HA-qCHI/pH6 was determined by measuring the relative viscosity of the supernatants of the complex coacervates formulated at 0.1 M NaCl with ratios (−):(+) ranging from 0.4 to 1.6. For the viscosity measurement, the flow time of the supernatants was measured by using a Ubbelohde capillary viscometer. The reported value is an average of three measurements which all showed less than ±1% variation. The relative viscosity was calculated from the ratio , where *t*_s_ is the flow time of the sample, and *t*_0_ is the flow time of the solvent (H_2_O + 0.1 M NaCl at pH 4 or pH 6). Any polymer chain in excess was assumed to remain in the supernatant phase, thus increasing its viscosity. Then, a minimum of the relative viscosity of the supernatant was considered as the optimum mixing ratio of the two polyelectrolytes for a given pH value. It is worth noting that under all conditions, the ratio (−):(+) = 1:1 was measured to be the optimum ratio for complex coacervation.

### 2.7 Complex Coacervate Preparation

Complex coacervates were all prepared at pH 4 or 6 using HA combined with CHI or synthesized qCHI with an equal concentration of positively and negatively charged units of 0.012 M. Stock solutions of HA, CHI, and qCHI at a concentration of 10 mg/mL were prepared at pH 4 and 6 according to the following procedure. CHI was first dispersed in DI water, then the pH of the dispersion was adjusted to 4 using a 1 M HCl solution to obtain a transparent homogeneous solution after 30 min of stirring. qCHI and HA were directly dissolved in deionized water and stirred for 30 min. The pH of the stock solutions was then adjusted to 4 or 6 using 1 M HCl and 1 M NaOH. Complex coacervates at different salt concentrations (0.0, 0.05, 0.1, 0.15, 0.2, 0.3, 0.4, 0.5, and 0.6 M NaCl) were prepared volumetrically by first mixing HA stock solutions of pH 4 or 6 with deionized water and 5 M NaCl solution followed by 30 s of vortex mixing and subsequent addition of the desired volume of CHI or qCHI stock solution at pH 4 or 6 (see Table S1 to S4 for the final compositions). Urea was added as a powder before CHI stock solution addition for the corresponding samples. The pH values of the mixtures remained unchanged after urea addition. The mixtures were then vortexed for 2 min and finally centrifuged at 4500*g* for 20 min at 20 °C. The total volume of all samples was 10 mL. The dense HA–CHI or HA–qCHI complex coacervate phase was left to rest overnight, then separated from the supernatant, and used as is for further studies.

### 2.8 Thermogravimetric Analysis (TGA)

A TGA Discovery series 5500TGA instrument was used to determine the composition of the complex coacervate samples. Around 5 mg of sample was subjected to a ramp of 20 °C/min from 20 to 700 °C under air flow without prior weight stabilization. The first weight loss until about 130 °C can be attributed to evaporation of water. The second weight loss between 200 and 600 °C is attributed to the organic HA and CHI polymer content. The remaining measured weight at 700 °C is attributed to the inorganic NaCl content. The results are expressed in weight percentage (wt %) compared to the original weight of the samples.

### 2.9 Phase Diagram Construction

The phase diagram of the HA–CHI and HA–qCHI systems was constructed by measuring the composition of both the supernatant and complex coacervate phases. The composition of the coacervate phase was obtained by TGA and is expressed as weight percentage of water, polymer (C_p_) and salt (C_s_). The salt content of the supernatant was measured by conductivity measurements using a calibration curve of the conductivity as a function of NaCl concentration. The polymer content in the supernatant was calculated by subtraction of the amount determined in the complex coacervate from the starting mass introduced in the mixture. It is important to notice that the low concentration of polyelectrolyte chains in the supernatant contributed to only a negligible extent to the overall conductivity of the supernatant. The results are reported as the mean value ± standard deviation of three independent sets of experiments for each sample.

### 2.10 Rheology

The linear viscoelasticity of the complex coacervates was determined by using small-amplitude oscillatory shear measurements on an Anton Paar MCR302e strain-controlled instrument. Strain amplitude measurements from 0.1 to 10% at a fixed angular frequency of 100 rad/s were first conducted to determine the linear viscoelastic region. Next, frequency sweeps were conducted over a range of frequencies from 100 to 0.1 rad/s at a fixed strain of 1%, well within the linear viscoelastic regime. HA–CHI samples prepared at pH 4 and HA–qCHI samples prepared at pH 4 and 6 were studied using a 25 mm diameter stainless steel cone–plate with a 1° angle (CP25–1). HA–CHI samples prepared at pH 6 were studied using a 10 mm diameter cross-hatched stainless steel plate (PP-10/S) to prevent wall-slip. All of the measurements were performed with a normal force below 0.1 N, reflecting fully relaxed systems. To prevent evaporation, 2 mL of the supernatant was poured around the geometry. The temperature was controlled via a Peltier cell connected to a recirculating bath and fixed to 20 °C for all the measurements.

### 2.11 Time–Salt–Superposition (TSS)

The method used to obtain the master curves from time–salt superposition is presented as follows. First, a Cole–Cole plot (*G*″ vs *G*′) as well as a van Gurp–Palmen plot (tan δ vs complex modulus G*) were done to qualitatively assess the feasibility of a TSS for a series of complex coacervate materials as a function of the salt concentration.^35,36,50^ Second, the horizontal shift factor, **a**_**s**,_ was determined from the plot of the loss factor, tan δ, as a function of the angular frequency, ω, taking the data from the sample prepared at 0.0 M NaCl as the reference. Third, the average vertical shift factor, **b**_s_, was obtained by vertically shifting the viscoelastic curves as a function of **a**_**s**_**ω** to match both the *G*′ and the *G*″ values (an averaged **b**_**s**_ was obtained from both *G*′ and *G*″ shifts) prepared at 0.0 M NaCl as the reference.

### 2.12 ^1^H NMR

^1^H NMR experiments in D_2_O were performed on a Bruker Avance III HD spectrometer operating at 400 MHz, using a standard 5 mm broadband Smart probe regulated at 25 °C. Chemical shifts are reported in parts per million from tetramethylsilane referenced to the residual isotopomer solvent signal (HOD).

### 2.13 UV–Visible Spectroscopy (UV–vis)

The optical transmittance measurements were carried out with a UV–Vis Hitachi U-1800 spectrophotometer using a quartz cell with a 1 cm path length. For the complex coacervate mixtures, the transmittance at 600 nm is reported as a function of the salt concentration just after the addition of the CHI stock solution to the solution containing HA and NaCl and thorough mixing. For the CHI and qCHI (1 mg/mL) solutions, the transmittance at 600 nm is reported as a function of the pH.

### 2.14 Small-Angle X-ray Scattering (SAXS) and Wide-Angle X-ray Scattering (WAXS)

SAXS and WAXS experiments were performed at the Multipurpose X-ray Instrument for Nanostructural Characterization (MINA) at the University of Groningen. The instrument is equipped with a high-intensity Cu rotating anode X-ray source, providing a parallel collimated X-ray beam with a photon wavelength of λ = 0.1543 nm. In order to explore a very broad q-range (0.05–8 nm^–1^), the SAXS data were acquired using two different sample-to-detector distances of 3 and 0.24 m, while the WAXS measurements were performed using a sample-to-detector distance of 0.08 m (q-range 8–20 nm^–1^). The scattering patterns were collected using a Bruker Vantec2000 detector (pixel size of 68 μm × 68 μm) and a Bruker Vantec500 detector (pixel size of 136 μm × 136 μm). The samples were prepared by loading the complex coacervate samples in a sealed glass capillary of 1.5 mm outer diameter with 0.01 mm wall thickness. The SAXS and WAXS patterns were converted into the 1D scattering intensity profiles by using Fit2D software. After subtracting the scattering signal from the solvent background, the three data sets were merged to generate the final SAXS/WAXS curves, where the scattering intensity profiles are plotted as a function of the modulus of the scattering vector q = 4πsin θ/λ. The sample-to-detector distance and the beam center position were calibrated by using the scattered rings from a standard silver behenate powder sample.

## 3 Results and Discussion

### 3.1 Phase Behavior and Linear Viscoelasticity of HA-CHI/pH4 and HA-CHI/pH6 Complex Coacervates

**Figure 1:**
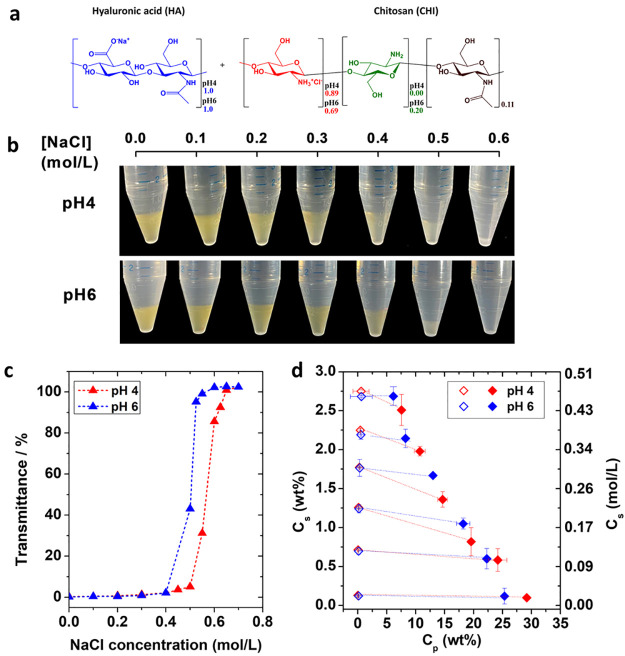
(a) Chemical structure of HA and CHI. The relative proportion of each repetitive unit is indicated for both pH 4 and pH 6. (b) Photographs of HA-CHI/pH4 (top) and HA-CHI/pH6 (bottom) samples from 0.0 to 0.6 M NaCl after centrifugation. (c) Evolution of the transmittance just after addition of the CHI solution to the HA solution as a function of the NaCl concentration at pH 4 and pH 6. (d) Phase diagram of the HA-CHI/pH4 and HA-CHI/pH6 systems. C_s_ and C_p_ are the salt content and the polymer content of the supernatant and complex coacervate phases, respectively. The open and closed marks correspond to the composition of the supernatant and the complex coacervate phase, respectively.

Here, our aim is to highlight the effect of pH at which HA–CHI complex coacervates are prepared on their phase and viscoelastic behavior. To this end, hyaluronic acid (*M*_w_ = 30–50 kg/mol) and chitosan (*M*_w_ = 30 kg/mol, degree of deacetylation *DDA* = 89%, Figure S1) were mixed together at NaCl concentrations ranging from 0.0 to 0.6 M and at pH 4 and 6. These pH values were chosen since the ionization degree of CHI decreases from 100 to 78% when pH increases from 4 to 6 while the carboxylic acid units from HA all stay charged (Figure 1a, Figure S2a, S2b, and S2c). In other words, all of the amino units of the CHI chains are protonated at pH 4 and are present as −NH_3_^+^, whereas at pH 6, 22% are deprotonated and present as −NH_2_. It is well-known that −NH_2_ groups readily act as hydrogen bond donors and/or acceptors, which is actually one of the reasons, with the presence of hydrophobic acetylated repetitive units, why chitosan can only dissolve in water at pH values below its p*K*a.^28,37^ The presence of H-bonds may therefore be expected to affect the properties of the resulting material.

The coacervates were formulated with an optimum negative to positive charge ratio ([−COO^–^]/[−NH_3_^+^]) that is determined as the ratio at which the minimum amount of polymer was detected in the supernatant. For this, the viscosity of the supernatant was measured as a function of the ratio of charges [−COO^–^]/[−NH_3_^+^] at pH 4 and 6 and at a given salt concentration of 0.1 M NaCl (Figure S3a). The optimum charge ratio was determined to be 1 in both cases. The decrease in ionization degree of CHI at pH 6 was included in the calculation of the ratios, and only charged units were counted. A charge ratio of 1 is commonly reported for a broad range of complex coacervate systems including the HA–CHI system.^33,38^

Subsequently, the phase behavior of the coacervates at different pH was investigated not only by visual observation but also by measuring the turbidity of the phase separated systems as a function of NaCl concentration to determine the salt resistance of the complex coacervates. Figure 1b shows the mixtures after centrifugation at pH 4 and pH 6. Phase separation with a dense complex coacervate was observed until 0.6 M NaCl for pH 4 and 0.5 M NaCl for pH 6. Above this salt concentration, the tubes appeared completely homogeneous, evidencing the absence of complexation and phase separation. The turbidimetry results presented in Figure 1c are in accordance with the visual observations as the transmittance reaches 100% for 0.55 M and 0.65 M NaCl at pH 6 and pH 4, respectively. As a matter of fact, by using CHI chains with a molecular weight closer to ours (50–190 kg/mol), Sun et al. observed a similar salt resistance value of 0.65 M at pH 4.5 in water.^26^

In addition to the salt resistance, a phase diagram can be constructed by determining the composition of the polymer-poor phase (the supernatant) and the polymer-rich phase (the complex coacervate) at varying salt concentrations. In brief, the composition of the coacervate phase was determined by thermogravimetric analysis (TGA) in which water, polymer, and salt contents could be distinguished. The composition of the polymer-poor phase was determined by a combination of conductometric and gravimetric measurements. More details concerning the construction of the phase diagrams shown in Figure 1d for the systems HA-CHI/pH4 and HA-CHI/pH6 can be found in the Experimental Section. For both pH conditions, the polymer content in the complex coacervates was found to decrease, while the water content increased with the added NaCl concentration. These trends are commonly observed for complex coacervate systems where salt acts as plasticizer.^21,38^ Moreover, Figure 1d shows that the polymer content in the complex coacervate phases is slightly lower for the HA-CHI/pH6 system compared to the HA-CHI/pH4 system for every NaCl concentration. Both observations of a lower salt resistance and a lower polymer content at higher pH may be explained by the change in charge density on the CHI chains (decreased by 22%) between pH 4 and pH 6: the higher the charge density, the higher the salt resistance and the higher the polymer content in the resulting complex coacervate. This is in agreement with results obtained by Neitzel et al., who studied the influence of the charge density in complex coacervates by incorporation of neutral monomers.^39^ The chemical nature, i.e., the water solubility, of the neutral units also influences the salt resistance and polymer content of the resulting complex coacervates. This will be discussed in more detail in section 3.2.

**Figure 2:**
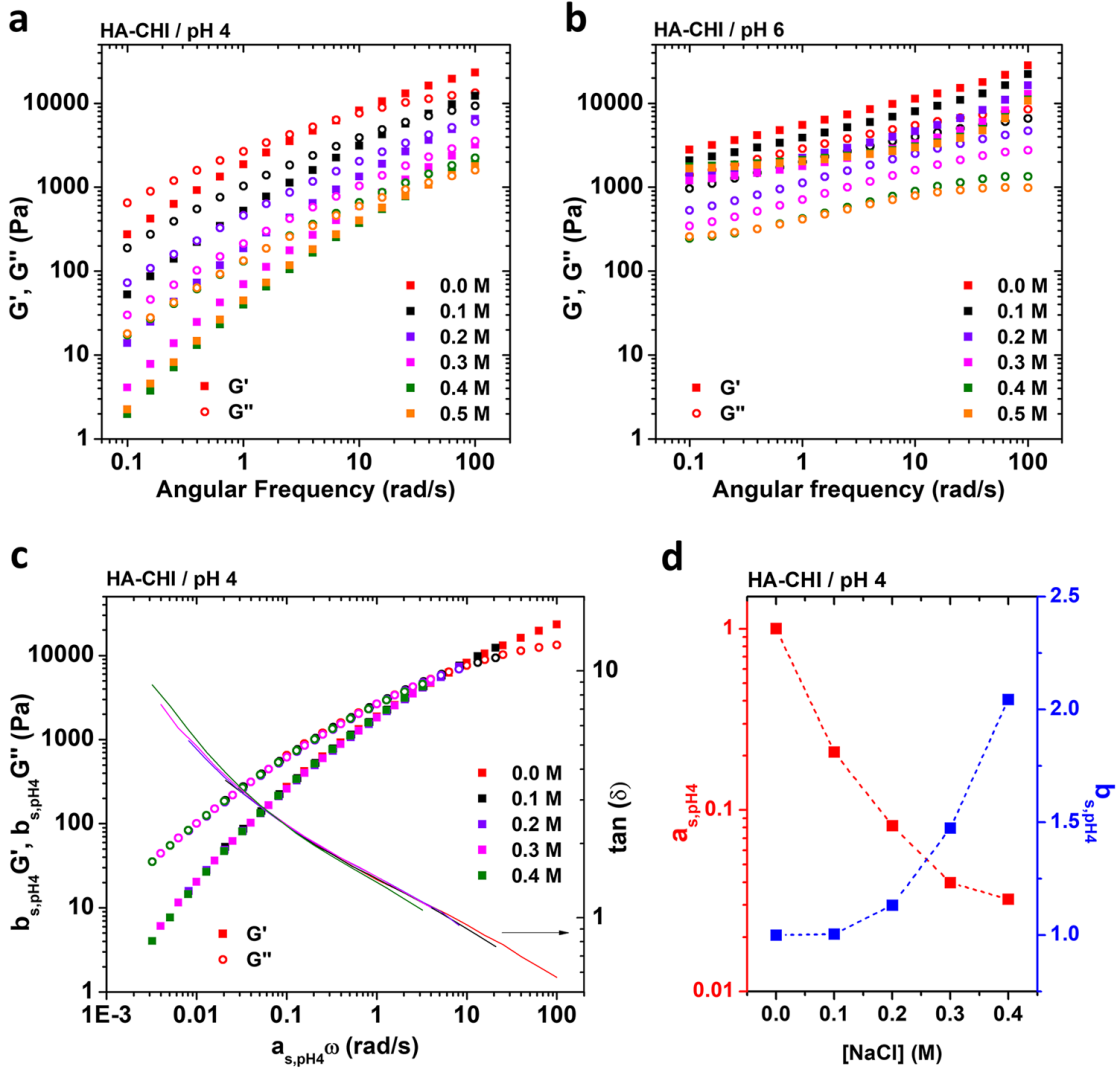
Frequency sweep data for (a) HA-CHI/pH4 and (b) HA-CHI/pH6 at different salt concentrations. (c) Effective time–salt superposition data for the HA-CHI/pH4 systems **b**_**s,pH4**_**G′**, **b**_**s,pH4**_**G″**, and tan δ are plotted as a function of **a**_**s,pH4**_ω. The data obtained for the samples prepared at 0.0 M NaCl are taken as reference. (d) Horizontal shift factor, **a**_**s,pH4**_, and vertical shift factor, **b**_**s,pH4**_, as a function of the NaCl concentration for the HA-CHI/pH4 samples.

We then focused on characterizing the linear viscoelasticity of the HA–CHI complex coacervates at pH 4 and 6 (Figure 2a, Figure 2b, Figure S4a and Figure S4b). Figure 2a shows the curves obtained for samples from 0.0 to 0.5 M NaCl at pH 4. In the probed frequency range, the rheological response of the investigated complex coacervates is that of viscoelastic liquids, with detectable terminal relaxation time estimated as the inverse frequency at the crossover between *G*′ and *G*″. This has been observed in a wide range of complex coacervates.^17,19,21,26^ For the samples formulated at 0.0, 0.1, and 0.2 M NaCl, the *G*′ and *G*″ crossover frequency increases with the salt concentration (Table S5), reflecting the acceleration of the relaxation dynamics, due to less effective electrostatic interactions between polymer chains. For 0.3 M NaCl and above, no crossover frequency could be determined in the range of frequencies investigated (0.1–100 rad/s). This plasticizing effect is a common feature observed for complex coacervates in which the strength and dynamics of the electrostatic interactions are directly controlled by the presence of electrolytes in the medium.^17,21,38^ It was demonstrated by Spruijt et al. that the dynamics of complex coacervates driven by electrostatic interactions can be modeled by the sticky Rouse model, in which the number of associating sites (stickers) are controlled by the salt concentration.^17^ Additionally, the weakening of the electrostatic interactions induced by doping with the salt leads to a lower polymer content (Figure 1c) and consequently a lower density of polymer in the material, which results in the lower moduli values.

For a complex coacervate system, a powerful way to unravel the salt-activated dynamics is the possibility of constructing a master curve through an effective time–salt superposition. Traditionally, van Gurp–Palmen (tan δ vs complex modulus G*) and Cole–Cole plots (*G*″ vs *G*′) are first employed to investigate the homogeneous nature of polymer melts and composites, with a semicircle shape implying absence of any heterogeneity in the material.^35,36,50^ Consequently, from Figure S5, we observe that the data of varying salt concentrations collapse on one another, implying the same relaxation modes for all salt concentrations. Then, we show that the HA-CHI/pH4 samples could be successfully assembled on a master curve through effective time–salt superposition (Figure 2c). The horizontal shift factor (**a**_**s,pHX**_) accounts for the speedup of the stress relaxation dynamics with increasing salt concentration, while the vertical shift factor (**b**_**s,pHX**_), accounts for the change in polymer density of the material that was determined as described in the Experimental Section. The data obtained for the sample prepared at 0.0 M was taken as reference, and the sample prepared at 0.5 M NaCl was disregarded from the superposition as it was situated close to the salt resistance value where large-scale microstructural fluctuations become particularly important (Figure 1a).^40^ The **a**_**s,pH4**_ and **b**_**s,pH4**_ values for each salt concentration are reported in Figure 2d. By increasing the salt concentration up to 0.4 M, a horizontal shift factor of **a**_**s,pH4**_ = 0.03 was necessary to account for the increased dynamics, while a vertical shift factor **b**_**s,pH4**_ = 2.04 was necessary to account for the drastic decrease of polymer concentration in the coacervate phase as shown in Figure 1c. The horizontal and vertical shift factors measured here are in close agreement with the ones reported by Sun et al. for a HA–CHI system investigated at pH 4.5 in water where the ionization degree of both polymers is close to 100% and of the same order of magnitude as the ones reported earlier for other complex coacervate systems made with synthetic polymers.^20,21,26,41^ The possibility of performing this time–salt superposition shows that the relaxation rates are affected by the presence of salt but not the relaxation modes. In other words, the sample remains self-similar regardless of the salt concentration investigated. It is important to note that the acceleration in dynamics observed upon increasing the salt concentration is the result of two effects: (1) a decrease in the average lifetime of the electrostatic associations; and (2) the decrease in polymer concentration in the complex coacervate.

When it comes to the HA-CHI/pH6 system, the observed results are drastically different. Figure 2b shows frequency sweep experiments for HA-CHI/pH6 complex coacervates from 0.0 to 0.5 M. All samples exhibit a relatively weak frequency dependence where *G*′ overcomes *G*″ for the whole range of frequencies investigated (0.1 to 100 rad/s), which is a characteristic of gels.^42^ However, the moduli did decrease with increasing salt concentration, which is the same trend as that observed at pH 4. As expected from the frequency sweep data, it was impossible to rescale the curves onto a common master curve through a time–salt superposition (Figure S5b). In other words, at pH 6, the dynamics of the HA–CHI coacervates are controlled not only by electrostatic interactions but also by relatively long-lived interactions with lifetimes longer than the ones probed by the rheological measurements performed here. A similar gel-like behavior for HA–CHI complex coacervates was recently observed by Kayitmazer et al. at pH = 6.7 when formulated with CHI chains with a *DDA* of 37% and 76%.^34^ However, when formulated with CHI chains with an intermediate *DDA* of 54%, the HA–CHI complex coacervate remained a viscoelastic liquid. The authors pointed out the intricate balance between blocky distribution of the hydrophobic acetyl units leading to the “freezing” of the complex coacervate dynamics and the decreased charge density along the CHI chains leading to shorter-lived electrostatic interactions. In our case, as the *DDA* of the CHI is unchanged (89%), we rather attribute the presence of these long-lived interactions to a relatively high density of H-bond associations involving deprotonated amino units, −NH_2_, which are formed as a result of the decrease in CHI affinity for water at pH 6.^31^ These high-density H-bond associations, possibly combined with the effect of the hydrophobic acetylated repetitive units, may form dynamically arrested domains within the complex coacervate. This hypothesis will be further discussed in the following sections. Prior to that discussion, an indication of the associative behavior of the CHI chains is already observed in viscosity measurements at pH 6 (and not at pH 4) for concentrations above the overlap concentration (Figure S6).

To conclude, the phase behavior of HA–CHI complex coacervates has been shown to be weakly affected by the pH of coacervation. At pH 4, the salt resistance and the polymer content in the coacervate were slightly higher than those at pH 6. However, the effect of the pH is most striking with respect to the viscoelasticity of the resulting materials: at pH 4, the coacervates behave as viscoelastic liquids whose dynamics is controlled by the NaCl concentration, i.e., a combination of the alteration in electrostatic interactions dynamics and in overall polymer concentration, whereas at pH 6, they exhibit salt-insensitive dynamics with a weak frequency dependence.

### 3.2 Phase Behavior and Linear Viscoelasticity of HA-qCHI/pH4 and HA-qCHI/pH6 Complex Coacervates

**Figure 3:**
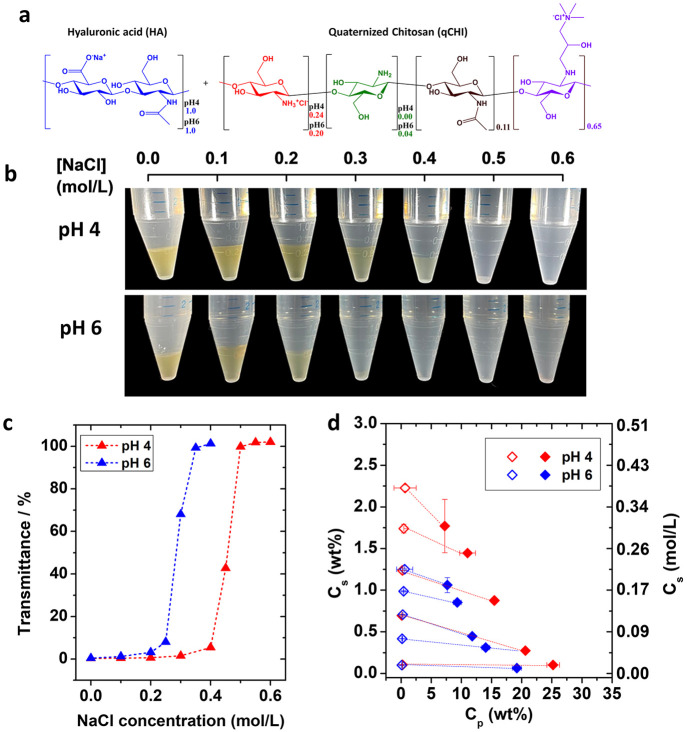
(a) Chemical structure of HA and qCHI. The relative proportion of each repetitive unit is indicated for both pH 4 and pH 6. (b) Photographs of HA-qCHI/pH4 (top) and HA-qCHI/pH6 (bottom) samples from 0.0 M NaCl to 0.6 M NaCl after centrifugation. (c) Evolution of the transmittance just after addition of the qCHI solution to the HA solution as a function of the NaCl concentration at pH 4 and pH 6. (d) Phase diagram of the HA-qCHI/pH4 and HA-qCHI/pH6 systems. C_s_ and C_p_ are, respectively, the salt content and the polymer content of the supernatant and complex coacervate phases. The open and closed marks correspond to the composition of the supernatant and the complex coacervate phase, respectively.

In this section, we aim to unravel the importance of intermolecular hydrogen bonding by investigating the complex coacervates made of HA and quaternized chitosan chains, qCHI, in which the charge of the quaternized repetitive units is independent of the pH of coacervation. For this, we performed a quaternization reaction of the CHI chains by grafting glycidyltrimethylammonium chloride moieties, GTMAC, on the amino units of the CHI chains (Figure S7). The resulting qCHI was characterized by ^1^H NMR spectroscopy (Figure S8) and the degree of quaternization (DQ), which corresponds to the number of primary amines turned into quaternary ammonium, was determined to be 72% by conductometric titration (Figure 3a and Figure S9). In striking contrast to CHI that precipitated at pH values above 6.4, the qCHI chains were fully soluble in water at least up to pH 12.2 (Figure S10), evidencing the successful increase of water solubility of the CHI chains. It has already been widely reported that quaternization weakens inter- and intramolecular hydrogen bonds and favors solubilization in water.^43,44^

As described in the previous section, we studied the phase behavior of HA–qCHI coacervates at pH 4 and pH 6. The optimum negative to positive charge ratio for complex coacervation of HA and qCHI, [−COO^–^]/[−NH_3_^+^] + [−N(CH_3_)_3_^+^], was also determined to be equal to 1 at both pH 4 and pH 6, identical to the native HA–CHI system (Figure S3b). Figure 3b shows the centrifuged HA-qCHI/pH4 and HA-qCHI/pH6 complex coacervates and Figure 3c the transmittance of the mixture upon addition of qCHI. As observed for the native HA–CHI system, the salt resistance was higher at pH 4 than at pH 6 (0.45 M vs 0.3 M NaCl) which is again attributed to the lower charge density on qCHI chains at pH 6.^39^ The phase diagrams of both HA-qCHI/pH4 and HA-qCHI/pH6 systems were also constructed and are presented in Figure 3d. Identical to the coacervates with unmodified CHI chains, the samples prepared at pH 4 contain a higher polymer content compared to the samples prepared at pH 6 at any given starting NaCl concentration. It could again be related to the difference in charge density of the qCHI chains even if 72% of the amino units are quaternized and are not pH-dependent anymore.

If we compare the samples with qCHI with samples with CHI we observe the following differences: (i) The salt resistance values of the HA–qCHI system were significantly lower than for the HA–CHI system, whether it is at pH 4 (0.4 M vs 0.6 M NaCl) or at pH 6 (0.2 M vs 0.5 M NaCl). (ii) The polymer content in the coacervate phase decreases for samples with qCHI instead of CHI at given pH and NaCl concentration. These differences can be explained by the increased hydrophilicity of the resulting qCHI chain backbone compared to the native CHI ones and also by the bulkiness of the GTMAC units. Previous studies have shown that bulky GTMAC units decrease the ability of the CHI chains to crystallize and then can impact the way the two polyelectrolytes interact with each other.^44^

Our observations are in agreement with studies that showed the opposite trend in the polymer concentration and salt resistance for increased hydrophobicity: Liu et al. specifically investigated the influence of the hydrophobicity of the backbone of synthetic strong polyelectrolytes containing sulfonate and ammonium groups on the phase behavior of the resulting complex coacervates.^41^ The authors measured a consistently higher polymer concentration as well as a higher salt resistance of coacervates formulated with methacrylated polymers compared with acrylated ones. They incriminated the hydrophobic methyl moieties of the methacrylated polymers as being the responsible factor. An identical observation was made by Tabandeh and Leon on polypeptides modified with an increased content of hydrophobic comonomer.^45^ Sadman et al. also showed that polyelectrolyte complexes of strong polyelectrolytes exhibited a higher resistance to doping with salt when the polymers were hydrophobically modified.^46^

**Figure 4:**
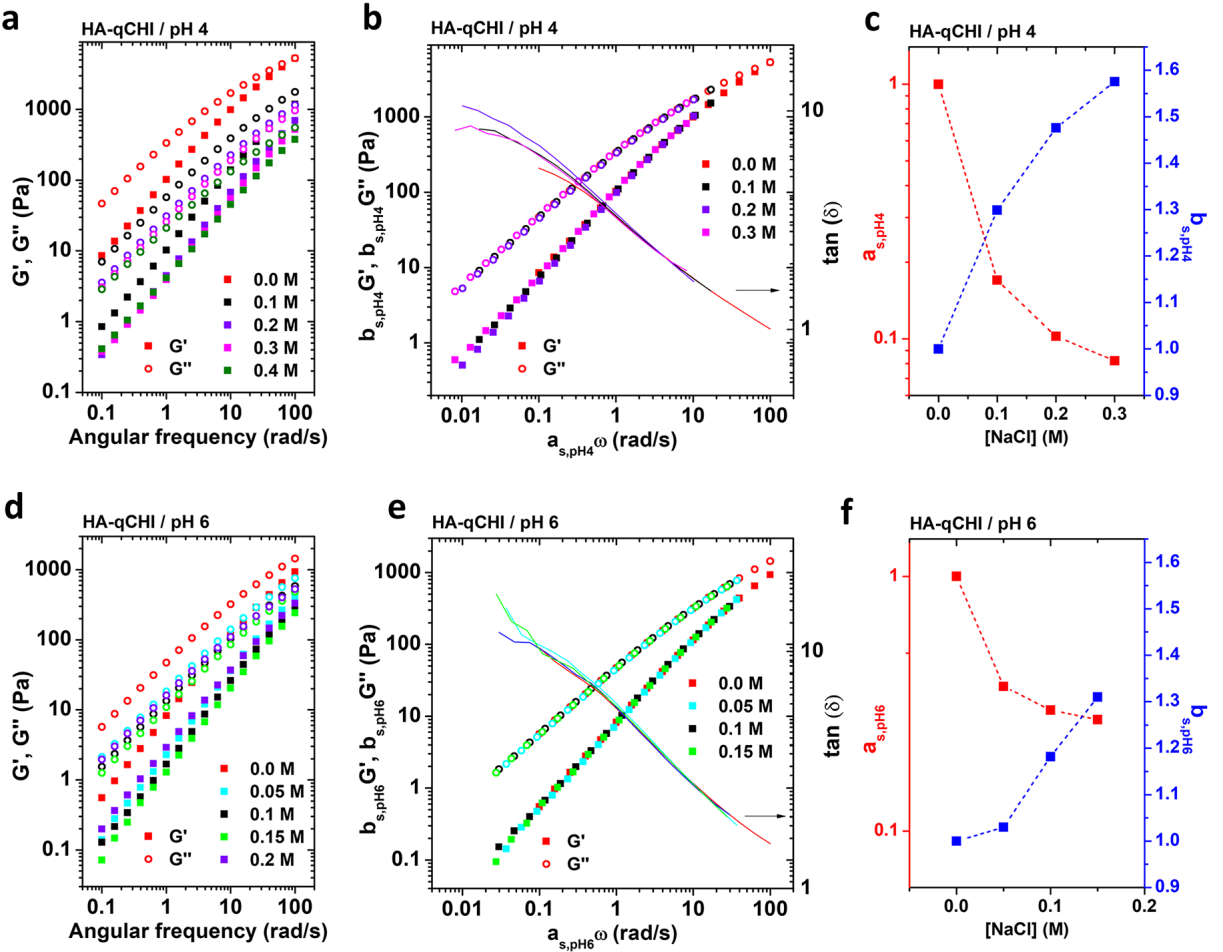
Frequency sweep data for (a) HA-qCHI/pH4 and (d) HA-qCHI/pH6 at different salt concentrations. Effective time–salt superposition data for (b) HA-qCHI/pH4 and (e) HA-qCHI/pH6. **b**_**s,pHX**_**G′**, **b**_**s,pHX**_**G″**, and tan δ are plotted as a function of **a**_**s,pHx**_ω. The data obtained for the samples prepared at 0.0 M NaCl are taken as reference. Horizontal shift factor, **a**_**s,pHX**_, and vertical shift factor, **b**_**s,pHX**_, as a function of the NaCl concentration for (c) HA-qCHI/pH4 and (f) HA-qCHI/pH6.

Following the same approach as for the HA–CHI couple, we investigated the linear viscoelasticity behavior of the HA–qCHI complexes at pH 4 and pH 6. The frequency sweep data for both the pH values are presented in Figure 4a and Figure 4d. Irrespective of the pH and the salt concentration investigated, all of the formulated HA–qCHI complex coacervates behave as viscoelastic liquids with *G*″ larger than *G*′ over the whole range of frequencies investigated. A closer inspection of the data obtained for the system HA-qCHI/pH4 reveals an increase in dynamics as well as a decrease of the moduli values by 1 order of magnitude as compared to the system HA-CHI/pH4. This difference can again be explained by the increased hydrophilicity of the qCHI chains, as already pointed out for the different phase behaviors of the two systems. An even more striking consequence of the quaternization of CHI lies in the tremendous difference in viscoelasticity at pH 6 between the HA–CHI and HA–qCHI couples. While the HA–CHI complex coacervates all behaved as elastic dynamically arrested gels from 0.0 to 0.5 M NaCl, the HA–qCHI complex coacervates are all viscoelastic liquids (from 0.0 to 0.2 M NaCl, which is the onset of phase separation). In addition, the moduli values are considerably lower in the latter case. For the HA–qCHI systems, effective time–salt superpositions were performed, and the data sets were plotted on a single master curve for both the HA-qCHI/pH4 system (Figure 4b) and the HA-qCHI/pH6 system (Figure 4e). It is worth noting that the deviations in superposition at lower frequencies are due to experimental limitations of the rheometer. The horizontal shift factors, **a**_**s,pHX**_, and the vertical shift factors, **b**_**s,pHX**_, with X being 4 or 6 depending on the pH, are reported in Figure 4c and Figure 4f. These results unambiguously show that the relaxation modes at pH 4 and pH 6 are identical and are activated by the salt concentration. Similar to the HA-CHI/pH4 system, the change in average lifetime of the electrostatic associations and in overall polymer concentration within the complex coacervate samples are the two effects that cause the change in stress relaxation dynamics. The self-similarity over a varying salt concentration is now ensured for the HA–qCHI system at pH 4 and maintained at pH 6.

### 3.3 Time–Salt–pH Superposition for the HA–qCHI System

**Figure 5:**
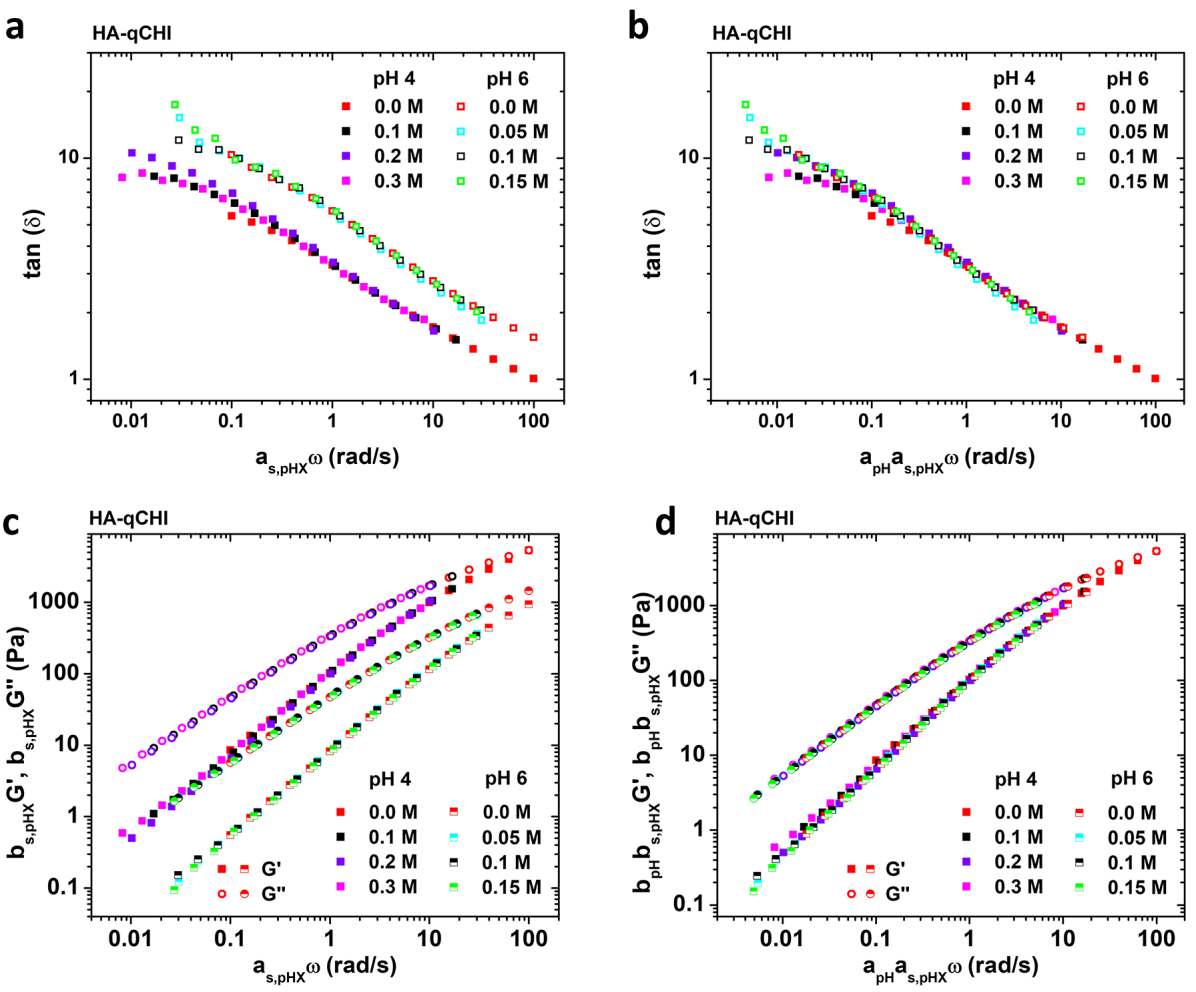
Effective time–salt superposition for the systems HA-qCHI/pH4 and HA-qCHI/pH6 showing (a) loss factor and (c) frequency sweep data as a function of **a**_**s,pHX**_ω. The samples prepared at 0.0 M NaCl are taken as reference. Effective time–salt superposition for the systems HA-qCHI/pH4 and HA-qCHI/pH6 showing (b) loss factor and (d) frequency sweep data as a function of **a**_**pH**_**× a**_**s,pHX**_. The data obtained for the samples prepared at pH 4 are taken as reference. The horizontal shift factor, **a**_**pH**_ = 0.17 and the vertical shift factor, **b**_**pH**_ = 1.62.

The results previously presented indicate salt-dependent dynamics for HA–qCHI for both pH 4 and pH 6. However, by plotting the loss factor and the shifted storage and loss moduli master curves on the same graph (Figure 5a and Figure 5c), we can observe that the curves do not overlap, suggesting a significant pH effect on the dynamics. Figure 5b and Figure 5d show that two master curves can be further rescaled onto a third one by shifting horizontally and vertically the HA-qCHI/pH6 with single shift factors **a**_**pH**_ = 0.17 and **b**_**pH**_ = 1.62. This horizontal shift factor, **a**_**pH**_, which is below 1, accounts for the slower dynamics observed at pH 4 compared to pH 6, while the vertical shift factor, **b**_**ph**_, which is above 1, indicates the lower polymer concentration in the complex coacervates at pH 6 compared to pH 4 for similar salt concentrations, in agreement with the phase diagram presented in Figure 3d. This successful shift of the viscoelastic data as a function of the pH unambiguously proves that the pH of the medium can affect the dynamics of the resulting HA–qCHI similar to the salt concentration. This time–pH superposition is only possible in the condition that the charge density in the complex coacervates made of the partially charged qCHI does not alter the self-similarity of the samples. In other words, the pH change only affects the time scale of the relaxation dynamics, but not the relaxation mechanisms. Tekaat et al. observed similar pH-dependent dynamics for complex coacervates made of the weak polyanion poly(acrylic acid), pAA, combined with the strong polycation poly(diallyldimethylammonium chloride), pDADMAc.^47^ The pH was varied in the range of 5 to 10, which corresponds to pAA ionization degrees from 0.4 to 1.0, while the pDADMAc ionization degree is unaffected and equal to 1.0 due to the strong nature of the quaternary ammonium cation. The authors also showed that the frequency sweep data obtained in this range of pH values at a fixed KCl concentration and charge ratio of 1 could be rescaled on a single master curve. Consequently, as stated previously, by quaternizing the CHI chains, the number and density of long-lived interactions leading to dynamically arrested domains are drastically reduced.

### 3.4 Effect of Dynamically Arrested Interactions on the Morphology of the HA–CHI Complex Coacervates

**Figure 6:**
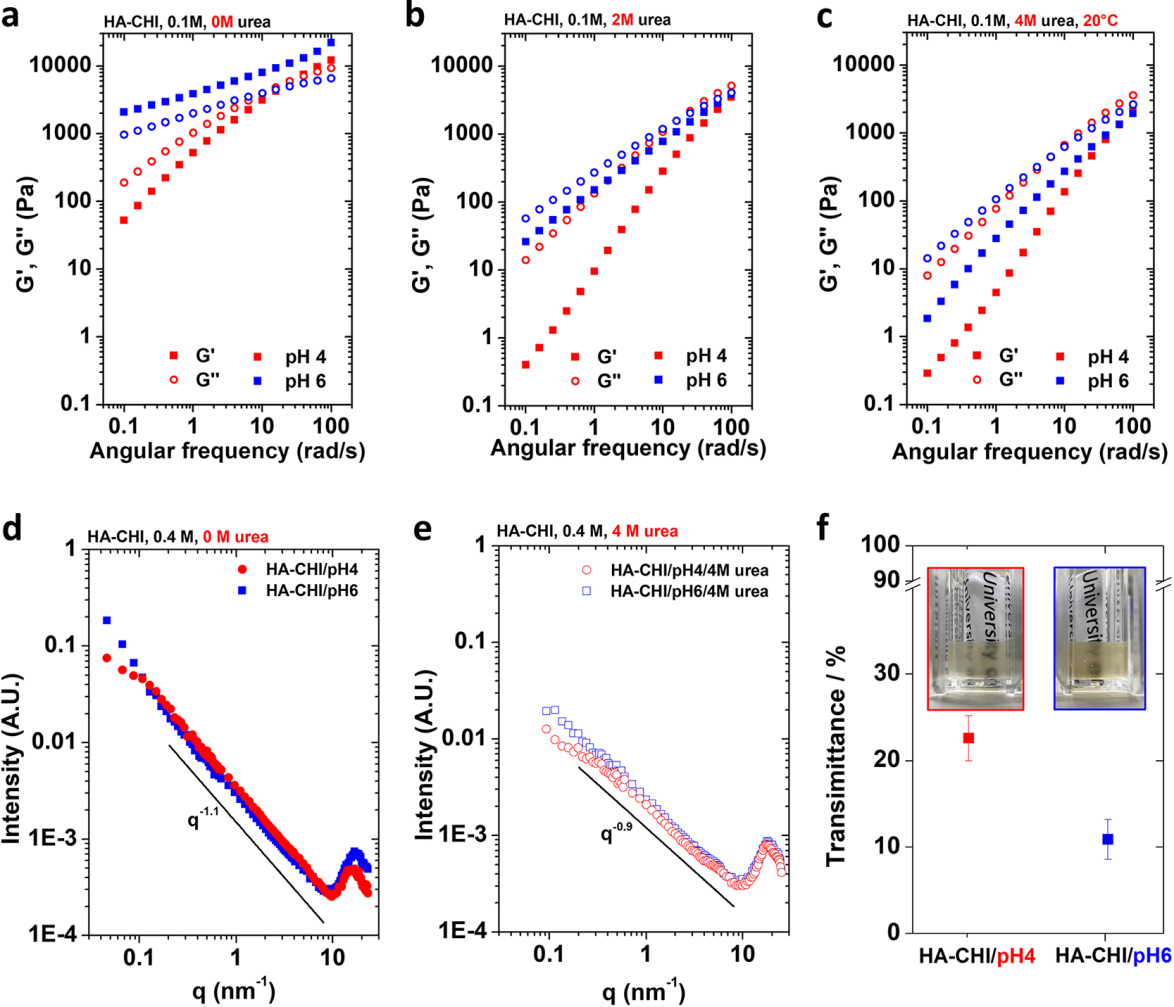
Rheological data of the HA-CHI/pH4 and HA-CHI/pH6 samples were analyzed at 0.1 M NaCl. Frequency sweep data with (a) 0 M, (b) 2 M urea, and (c) 4 M urea. SAXS–WAXS intensity profiles for the HA-CHI/pH4 and HA-CHI/pH6 complex coacervates at 0.4 M NaCl formulated (d) without urea and (e) with 4 M urea. (f) Absorbance of the HA-CHI/pH4 and HA-CHI/pH6 complex coacervates at 0.4 M NaCl determined by UV–vis spectroscopy. The insets are the photographs of the corresponding the samples.

To shed light on the presence of additional dynamically arrested domains at pH 6 in the HA–CHI complex coacervates, we first address the effect of urea (an efficient hydrogen bond competitor) on the dynamics of the resulting materials.^27^ The viscoelastic behavior of the HA-CHI/pH4 and HA-CHI/pH6 samples at 20 °C and 0.1 M NaCl formulated with 0, 2, and 4 M urea is shown in Figure 6a, 6b, and 6c. We can clearly observe that increasing the concentration of urea in the systems renders the HA–CHI complex coacervates more fluid-like, with *G*″ overpassing *G*′ over the whole range of frequencies investigated, whether it is at pH 4 or pH 6. The most striking observation is that at 4 M urea, the frequency response of HA-CHI/pH4 and HA-CHI/pH6 samples almost completely overlap. This shows the potential of urea to disrupt the extra hydrogen-bonded, dynamically arrested domains present at pH 6, transitioning the sample from an elastic solid-like material to a viscous liquid.

Second, SAXS and WAXS experiments were carried out to reveal the microstructure of the HA–CHI complex coacervates at pH 4 and pH 6 and 0.4 M NaCl (Figure 6d) and to elucidate the effect of the urea addition (Figure 6e). The total scattering curve for the HA–CHI complex can be divided into three regions: a low-angle SAXS region (*q* < 0.2 nm^–1^), a mid-angle SAXS region (0.2 nm^–1^ < *q* < 4 nm^–1^), and a high-angle WAXS region (*q* > 4 nm^–1^). In the mid-angle region, the SAXS profiles for the two complex coacervates show similar power-law behavior with q^–α^ with α ∼ 1.1. The value of the exponent is very close to 1, expected for rod-like objects, revealing that both samples are constituted by semi rigid polymeric bundles. Interestingly, the samples prepared at different pH show a marked difference at low scattering angles. While at pH 4, SAXS shows the presence of an intensity kink located at q ∼ 0.11 nm^–1^ that can be associated with the presence of aggregates with characteristic average size close to 50–60 nm and some possible degree of weak aggregation, at pH 6, the complex coacervate shows a clear intensity upturn, characteristic for the presence of a structure with a high level of branching and interconnections, directly related to the increase in the density of hydrogen bonds in the network, which explains the change in mechanical properties observed in the rheological data.^48,49^ The characteristic size of these interconnected structures is well above the resolution of the SAXS measurements (>130 nm), in agreement with turbidimetry measurements in which the HA-CHI/pH6 scatters significantly more light than HA-CHI/pH4 (Figure 6f). Some structural differences for the different pH values of preparation can also be extracted from the WAXS region. The complex coacervate prepared at pH 6 shows a more intense WAXS peak with an average peak position shifted toward higher scattering angles, i.e. shorter distances (16.1 nm^–1^ at pH 4 and 17.5 nm^–1^ at pH 6, respectively related to an average spacing of 0.39 and 0.36 nm). Since the internal arrangement of these coacervates is mostly amorphous, the WAXS peak is quite broad and is generated by the overlap of different contributions, both interchain (HA-to-CHI aggregation) and intrachain (monomer–monomer distances within the same chain, i.e., local conformation). Also, the change in the distribution of structural water around the polymer chains may affect the peak intensity. Despite it being not possible to isolate a single contribution, the shift of the WAXS peak at pH 6 could point toward a tighter association of the polymeric chains due to the larger density of hydrogen bonding. In a study aimed at understanding the mechanical properties of HA–CHI polyelectrolyte complexes, Lalevée et al. investigated the microstructure of their gels as a function of pH by means of SANS.^31^ The authors observed the presence of high-density zones at pH 6 that were absent at pH 2.5. Their average size was determined to be around 60–80 Å, and they assigned them as phase-separated CHI crystallite inclusions. However, the crystalline domains were reported not to be observed by SAXS due to negligible contrast. We note that in our data, at pH 6, a very weak bump located in the q-region between 0.5 and 2 nm^–1^ is present, which could be ascribed to some local ordering of the chains in agreement to Laleveé et al. Upon the addition of 4 M urea, the SAXS intensity of both coacervates at pH 4 and pH 6 drops by almost 1 order of magnitude, and the curves tend to flatten at larger angles with respect to the samples without urea, especially at pH 4 (Figure 6e). This is the result of a decreased stiffness of the polymeric bundles and a decreased degree of their association, which agrees well with the transition to a liquid-like behavior measured by rheology upon urea addition. Moreover, the presence of urea in the complex and the disruption of the extra hydrogen-bonded, dynamically arrested domains present at pH 6 also result in a very similar local structure between the two HA–CHI samples prepared at the different pHs, as evidenced by the overlapping WAXS signal. Additionally, the observed shift of the WAXS peak toward higher q-values for the samples containing urea both at pH 4 and at pH 6 compared to the samples prepared without urea may arise from tight hydrogen bonding interactions between the chains and the urea molecules which are abundant in the system.

**Figure 7:**
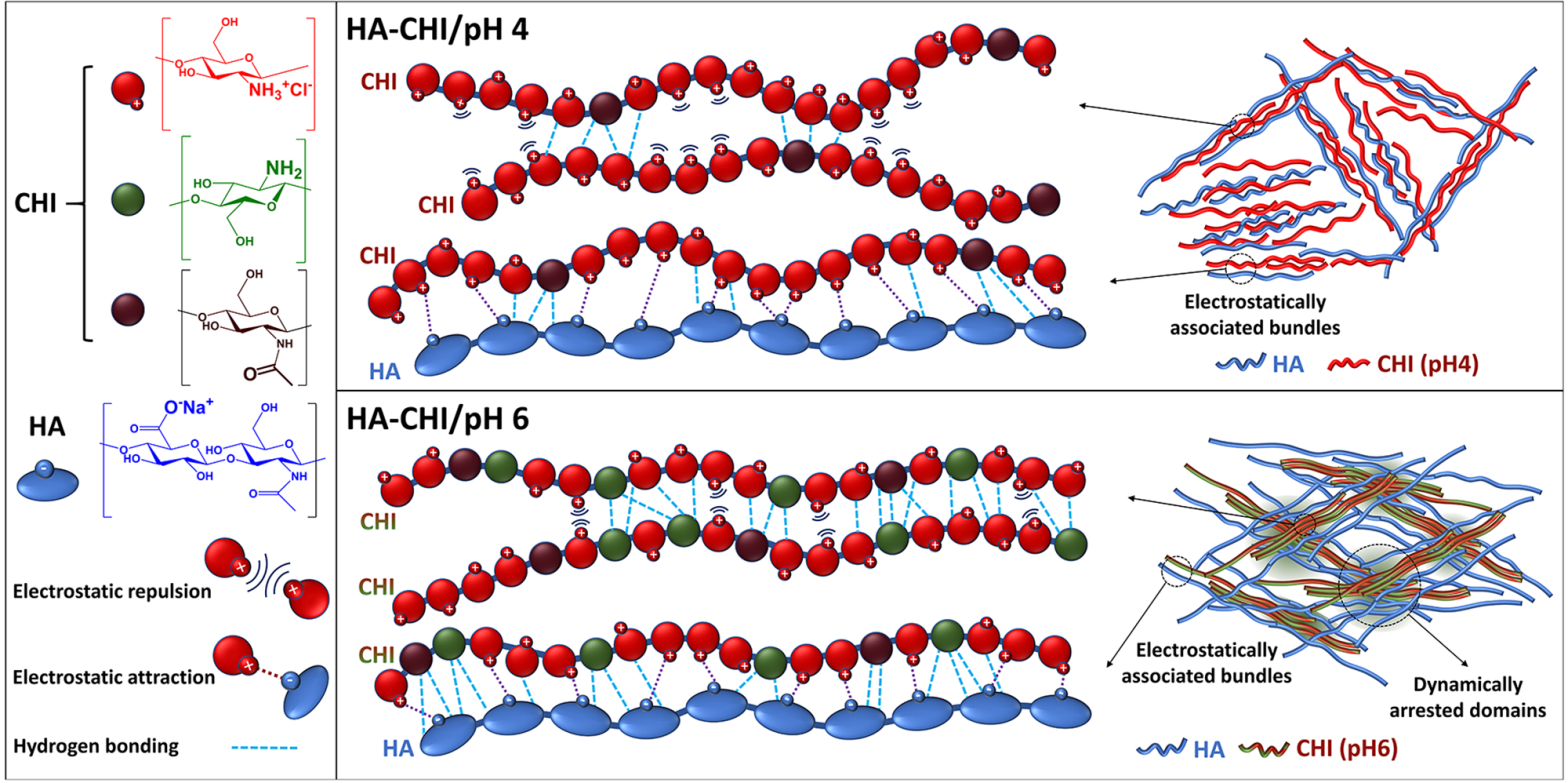
Schematic representation of the intermolecular interactions between CHI chains and HA and CHI chains and of the structuration of the HA–CHI complex coacervates at pH 4 and pH 6.

Altogether, these results point to the strong influence of intermolecular hydrogen bond associations inside the complex coacervates at pH 6 on the final microstructure and dynamics of the resulting material. Figure 7 shows the proposed structural scheme, in which the key differences between the HA-CHI/pH4 and HA-CHI/pH6 systems are highlighted. It is worth mentioning that the highlighted intermolecular associations between CHI chains do not prevent their full solubilization at both pH 4 and pH 6.

## 4 Conclusion

In summary, we bring to light the crucial effect of intermolecular hydrogen bonds on the phase behavior, viscoelastic properties, and microstructure of hyaluronic acid–chitosan complex coacervates. When formulated at pH 4, a pH for which the ionization degree of both polyelectrolytes is close to 1, the resulting complex coacervates behaved as viscoelastic liquids with increased dynamics and water content as the salt concentration was increased. An effective time–salt superposition master curve could be drawn from the viscoelastic data, evidencing the salt-activated dynamics of the coacervates formed. However, for the samples formulated at pH 6, a pH for which the primary amine units of the chitosan chains are only partially protonated, the resulting complex coacervates behaved as an elastic gel irrespective of the salt concentration investigated. For both pH values, the phase diagram showed an expected shape for complex coacervates with a decrease in the polymer content in the coacervate phase upon increasing the added salt concentration. In addition, a small but consistent decrease in the polymer content was evidenced by changing the pH from 4 to 6, which was assigned to the decrease of the charge density of the chitosan chains at pH 6, in accordance with literature.

Upon quaternization of the primary amine units of the chitosan chains, which ensured the solubility of the individual chains up to pH 12, the salt-activated dynamics behavior at pH 6 was recovered. The resulting complex coacervates behaved as a viscoelastic liquid over the whole range of frequencies investigated at either pH 4 or pH 6. Similar to the native system at pH 4, a successful master curve could be obtained for the series prepared at both pH 4 and pH 6. Furthermore, a time–salt–pH equivalence could be revealed for the hyaluronic acid–quaternized chitosan system as both master curves at pH 4 and pH 6 could be rescaled on an individual third master curve. Both the salt- and pH-activated dynamics for the latter system indicate that the sample remained self-similar in these conditions in striking contrast to the hyaluronic acid–chitosan system at pH 6.

In the last part, we investigated the effect of the presence of urea, a powerful hydrogen bond competitor, on the rheological behavior of the hyaluronic acid–chitosan coacervates. With the introduction of urea, we were able to disrupt the added hydrogen bonds in the system at pH 6 and to obtain identical viscoelastic behavior between the coacervates at pH 4 and pH 6. X-ray scattering experiments were performed to reveal the microstructure of the system as a function of the pH. SAXS and WAXS data revealed the presence of elongated interconnected polymeric aggregates for both hyaluronic acid–chitosan complex coacervates at pH 4 and pH 6, with a higher degree of association and branching for the denser polymeric network formed at pH 6. In the presence of urea, at both pH 4 and 6, the characteristic length of the aggregates drastically decreased, indicating a higher overall flexibility in direct correlation with the observed viscoelastic liquid behavior.

Our study shows the importance of the polymer–solvent affinity inside a complex coacervate resulting from a significantly higher density of intermolecular hydrogen bond interactions inside the matrix of the electrostatically associated polymer chains. The subsequent rheological signature can be drastically changed by a relatively limited modification of the pH conditions while barely affecting the composition of the material. This study paves the way to develop a rational design approach of complex coacervates for which solidification can be controlled by a slight change of physicochemical conditions.

## Supporting Information Available

^1^H NMR of CHI, Potentiometric titration and degree of ionization as a function of the pH of HA and CHI, Viscosity of the supernatant of the HA–CHI and HA–qCHI complex coacervates as a function of the charge ratio, Linear viscoelastic domain determination for the HA–CHI and HA–qCHI complex coacervates, Cole–Cole and van Gurp–Palmen plots for the HA–CHI and HA–qCHI complex coacervates, Overlap concentration of HA and CHI at pH 4 and pH 6, Reaction scheme for the qCHI synthesis, ^1^H NMR of qCHI, Conductometric titration of qCHI, Solubility of qCHI and CHI in water, Composition of the mixtures for the HA–CHI and HA–qCHI complex coacervates, Crossover frequencies of the HA-CHI/pH4 and HA-CHI/pH6 systems. (PDF)
