# Supplementary material for: Effect of Dynamically Arrested Domains on the Phase Behavior, Linear Viscoelasticity and Microstructure of Hyaluronic Acid – Chitosan Complex Coacervates
Source: Macromolecules. 2023 Jul 18;56(15):5891–904. doi: 10.1021/acs.macromol.3c00269 (PMC10413963; doi:10.1021/acs.macromol.3c00269)
Supplement: Supplementary file 1 — ma3c00269_si_001.pdf [file ma3c00269_si_001.pdf]

Supporting Information for

Effect of Dynamically Arrested Domains on the Phase Behavior, Linear Viscoelasticity and Microstructure of Hyaluronic Acid – Chitosan Complex Coacervates

Julien Es Sayed[a], Clément Caïto[a], Abinaya Arunachalam[a], Armin Amirsadeghi[a], Larissa van Westerveld[a], Denise Maret[a], Roshan Akdar Mohamed Yunus[b], Eleonora Calicchia[a,c], Olivia Dittberner[a], Giuseppe Portale[a], Daniele Parisi[b], Marleen Kamperman[a]*

[a]*Zernike Institute for Advanced Materials (ZIAM), University of Groningen, Nijenborgh 4, 9747 AG Groningen, the Netherlands.*

[b]*Engineering and Technology Institute Groningen (ENTEG), University of Groningen, Nijenborgh 4, 9747 AG Groningen, The Netherlands*

[c]*Department of Nanomedicine & Drug Targeting, Groningen Research Institute of Pharmacy, University of Groningen, A. Deusinglaan 1, 9713 AV Groningen, The Netherlands*

*E-mail: marleen.kamperman@rug.nl

^{1}H NMR characterization of CHI.

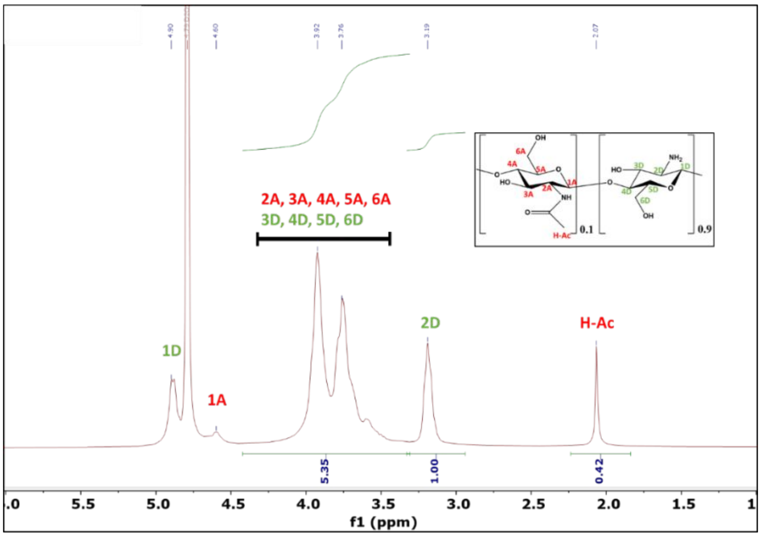

Figure S1. ^{1}H NMR spectrum (D_2O, 0.01 M DCl) of CHI. The degree of deacetylation (DDA) was measured by comparing the relative integration value of the H-Ac and the 2D peaks. DDA = DA =1/(1+ 0.40/3) = 1/ 1.13 = 89 %.

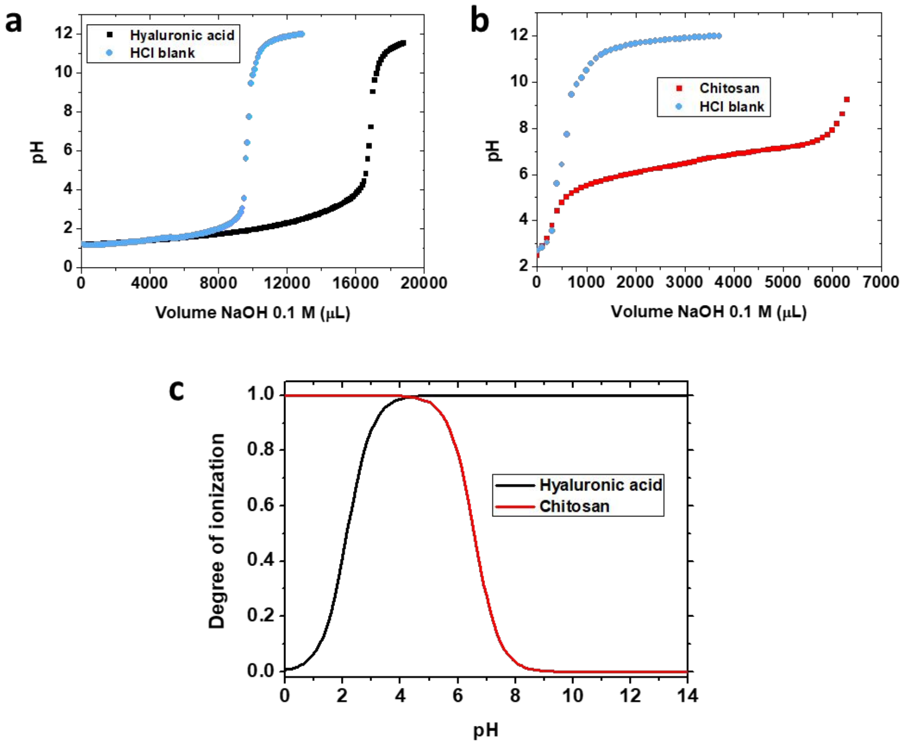

Figure S2. pH titration of **(a)** HA and **(b)** CHI. In both graphs the blank titration of excess HCl is represented with blue symbols. **(c)** Degree of ionization of HA and CHI as a function of the pH.

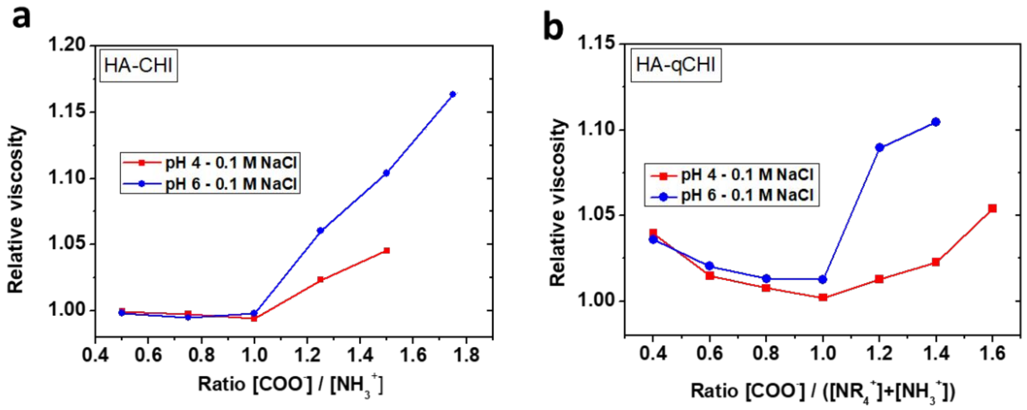

Figure S3. Relative viscosity of the supernatant of **(a)** HA-CHI and **(b)** HA-qCHI coacervates at 0.1 M NaCl at pH 4 and 6.

Determination of the linear viscoelastic regime.

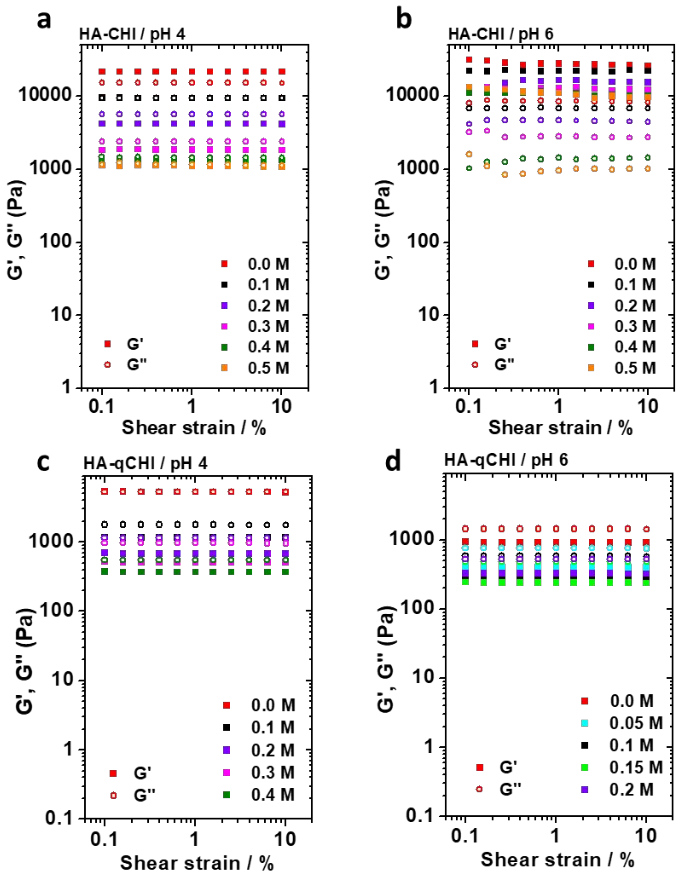

Figure S4. Amplitude sweep (0.1 – 10% strain at 100 rad/s) for the **(a)** HA-CHI/pH4, **(b)** HA-CHI/pH6, **(c)** HA-qCHI/pH4 and **(d)** HA-qCHI/pH6 samples.

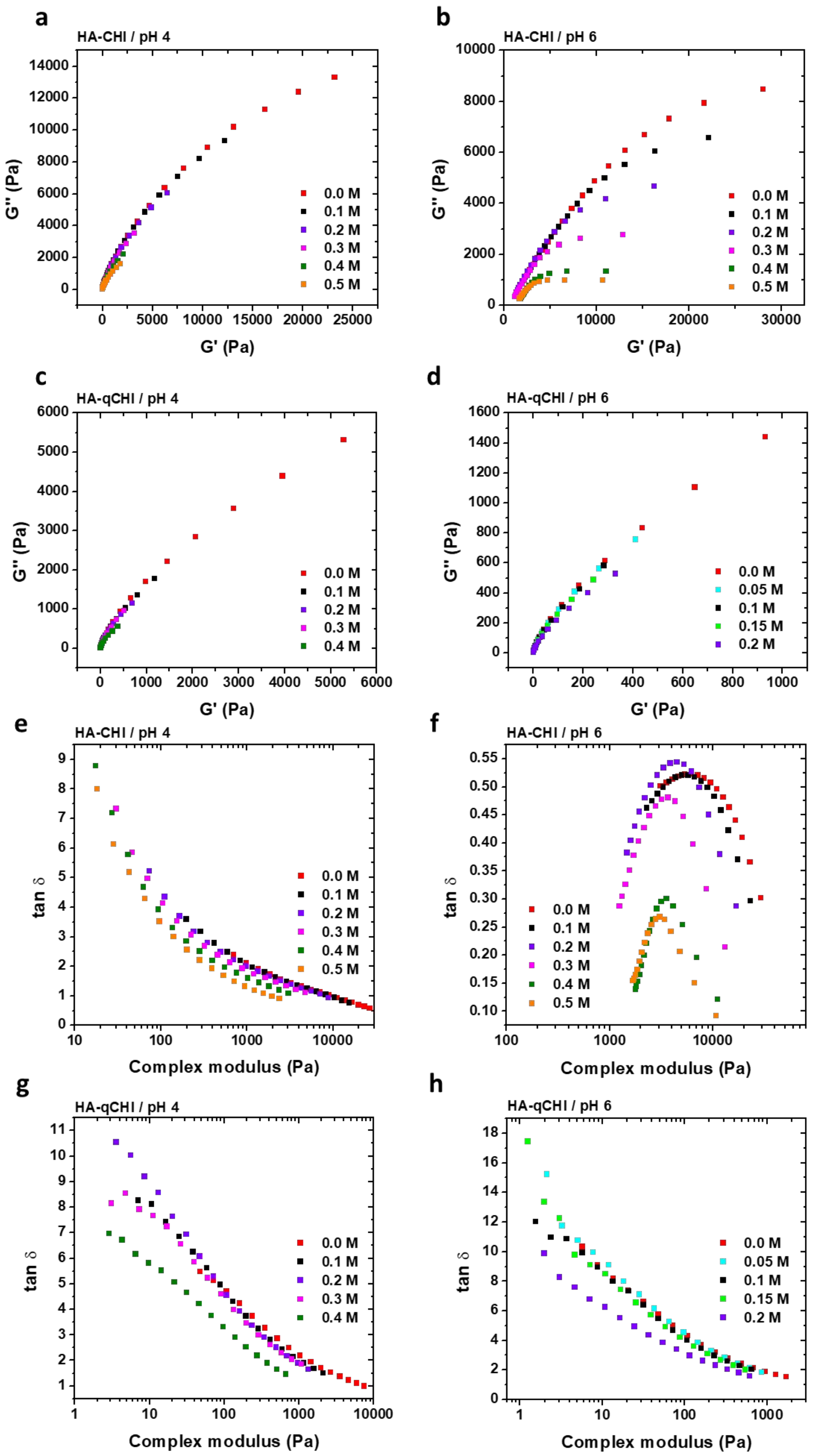

Figure S5. Cole-Cole plots for the **(a)** HA-CHI/pH4, **(b)** HA-CHI/pH6, **(c)** HA-qCHI/pH4 and **(d)** HA-qCHI/pH6 samples. Van Gurp - Palmen plots for the **(e)** HA-CHI/pH4, **(f)** HA-CHI/pH6, **(g)** HA-qCHI/pH4 and **(h)** HA-qCHI/pH6 samples.

Determination of the overlap concentration.

The evolution of the relative viscosity of CHI solutions at pH 4 and pH 6 as a function of the CHI concentration (**Figure S6**). For both pH conditions, the slopes in the dilute region were close. The overlap concentration were also found to be relatively close with C*pH4,CHI = 2.6 wt% and C*pH6,CHI = 2.2 wt% respectively. However, in the semi-dilute region the slope of the CHI at pH 6 was found to be drastically higher than at pH 4. This unambiguously proves that intermolecular associations, possibly arising from H-bonds, are present at pH 6 but not at pH 4 at concentrations above the overlap concentration. The overlap concentration was determined to be C*pH6,HA = 2.7 wt% in the same way.

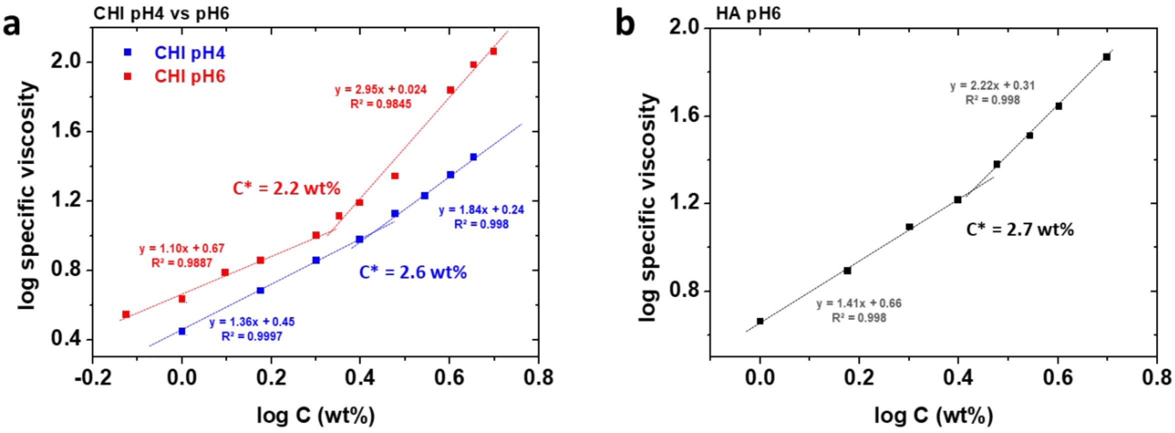

Figure S6. Overlap concentration measurement of **(a)** CHI at pH 4 and pH 6 and **(b)** HA at pH 6.

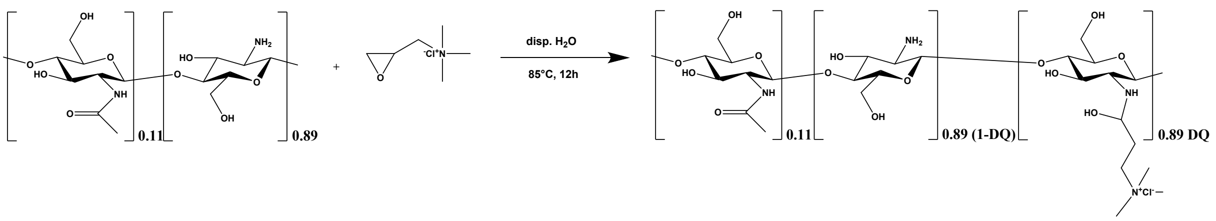

Figure S7. Reaction scheme for the synthesis of qCHI.

^{1}H NMR characterization of qCHI.

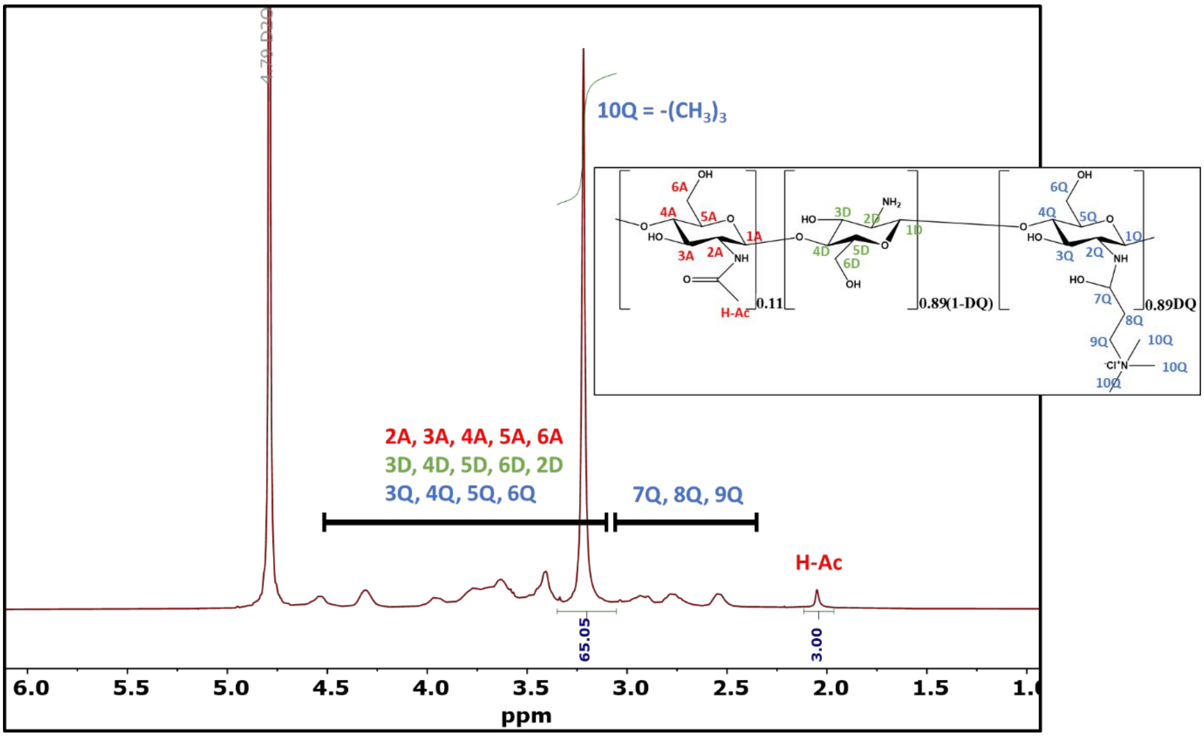

Figure S8. ^{1}H NMR spectrum (D_2O) of qCHI. The degree of quaternization (DQ) could not be obtained by this method. It is worth mentioning that only a qualitative proof of successful quaternization can be shown by the presence of the $–(CH_3)_3$ peak of the GTMAC units at 3.25 ppm. Indeed, the overlap of this peak with the ill-defined peaks from the CHI backbone spreading from 1.5 to 4.75 ppm renders the quantification of the DQ inaccurate.

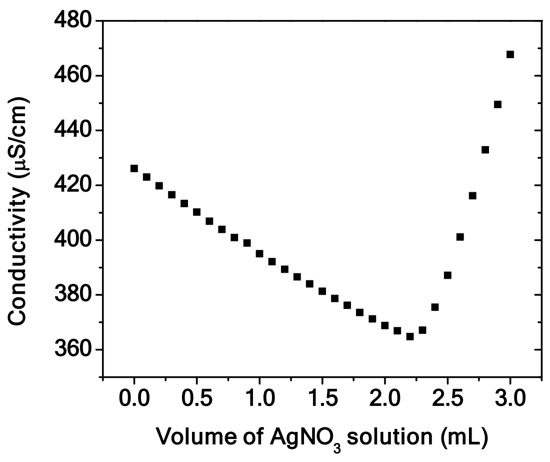

Figure S9. Conductometric titration of a 1 mg/mL solution of qCHI with $AgNO_3$.

The equivalent volume was determined to be $V(AgNO_3) = 2.23$ mL. From this, the degree of quaternization of the primary amines was calculated to be DQ = 72%.

qCHI vs CHI solubility in water.

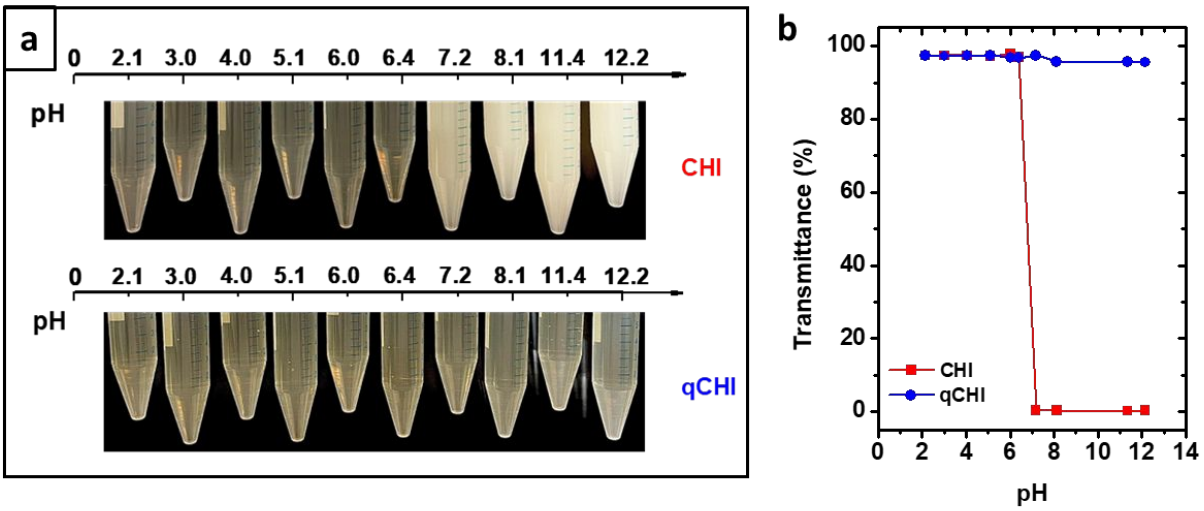

Figure S10. (a) Visual aspect of 1 mg/mL CHI (top) and qCHI (bottom) solutions in water as a function of the pH. **(b)** Evolution of the transmittance of 1 mg/mL CHI and qCHI solutions in water as a function of the pH. The CHI chains start precipitating above pH 6.4 while qCHI chains remain soluble on the whole range of pH investigated (from 2.1 to 12.2).

Table S1. Composition of the mixtures to form HA-CHI/pH 4 complex coacervates.

[NaCl] (mol/L)	Vtot (mL)	V HA (mL)	V NaCl 5M (mL)	V H_2O (mL)	V CHI (mL)
**0.0**	10.00	4.81	0	2.98	2.20
**0.1**	10.00	4.81	0.2	2.78	2.20
**0.2**	10.00	4.81	0.4	2.58	2.20
**0.3**	10.00	4.81	0.6	2.38	2.20
**0.4**	10.00	4.81	0.8	2.18	2.20
**0.5**	10.00	4.81	1	1.98	2.20
**0.6**	10.00	4.81	1.2	1.78	2.20

Table S2. Composition of the mixtures to form HA-CHI/pH 6 complex coacervates.

[NaCl] (mol/L)	Vtot (mL)	V HA (mL)	V NaCl 5M (mL)	V H_2O (mL)	V CHI (mL)
**0.0**	10.00	4.81	0	2.36	2.83
**0.1**	10.00	4.81	0.2	2.16	2.83
**0.2**	10.00	4.81	0.4	1.96	2.83
**0.3**	10.00	4.81	0.6	1.76	2.83
**0.4**	10.00	4.81	0.8	1.56	2.83
**0.5**	10.00	4.81	1	1.36	2.83
**0.6**	10.00	4.81	1.2	1.16	2.83

Table S3. Composition of the mixtures to form HA-qCHI/pH 4 complex coacervates.

[NaCl] (mol/L)	Vtot (mL)	V HA (mL)	V NaCl 5M (mL)	V H_2O (mL)	V qCHI (mL)
**0.0**	10.00	4.81	0	1.67	3.51
**0.1**	10.00	4.81	0.2	1.47	3.51
**0.2**	10.00	4.81	0.4	1.27	3.51
**0.3**	10.00	4.81	0.6	1.07	3.51
**0.4**	10.00	4.81	0.8	0.87	3.51
**0.5**	10.00	4.81	1	0.67	3.51
**0.6**	10.00	4.81	1.2	0.47	3.51

Table S4. Composition of the mixtures to form HA-qCHI/pH 6 complex coacervates.

**[NaCl] (mol/L)**	Vtot (mL)	V HA (mL)	V NaCl 5M (mL)	V H_2O (mL)	V qCHI (mL)
**0.0**	10.00	4.81	0	1.44	3.74
**0.05**	10.00	4.81	0.1	1.34	3.74
**0.1**	10.00	4.81	0.2	1.24	3.74
**0.15**	10.00	4.81	0.3	1.14	3.74
**0.2**	10.00	4.81	0.4	1.04	3.74
**0.3**	10.00	4.81	0.6	0.84	3.74
**0.4**	10.00	4.81	0.8	0.64	3.74
**0.5**	10.00	4.81	1	0.44	3.74
**0.6**	10.00	4.81	1.2	0.24	3.74

Table S5. Crossover frequencies determined by rheology for the HA-CHI/pH4 and HA-CHI/pH6 complex coacervates.

	**Crossover frequency (rad/s)**	
**[NaCl] (mol/L)**	**HA-CHI/pH4**	**HA-CHI/pH6**
**0.0**	≈ 8	< 0.1
**0.1**	≈ 32	< 0.1
**0.2**	≈ 81	< 0.1
**0.3**	> 100	< 0.1
**0.4**	> 100	< 0.1
**0.5**	> 100	< 0.1
